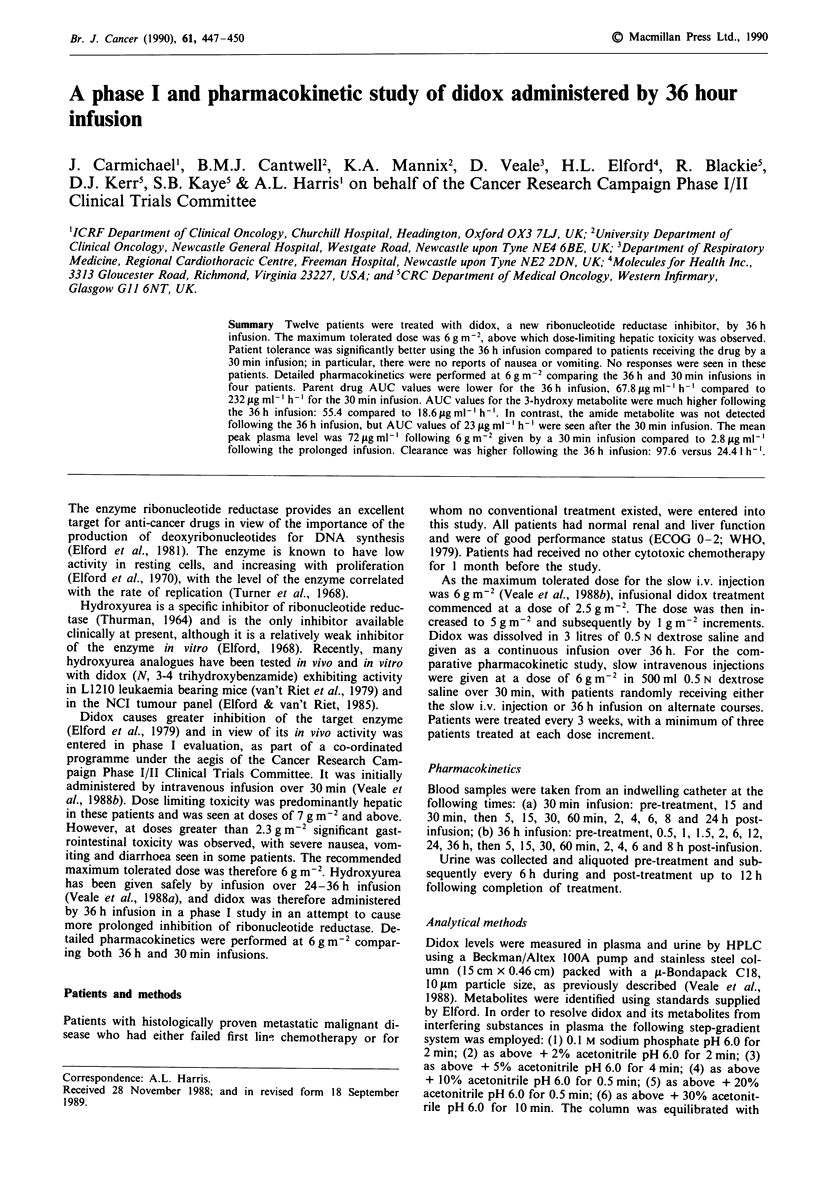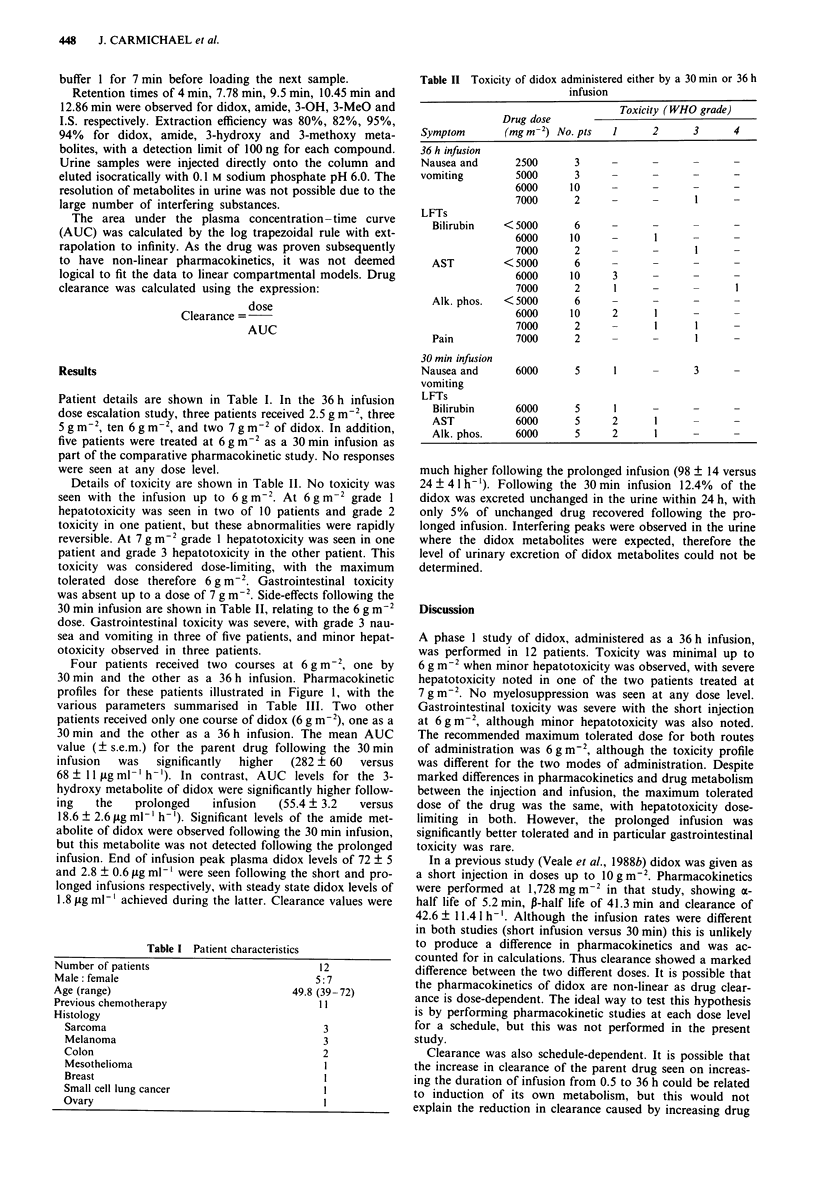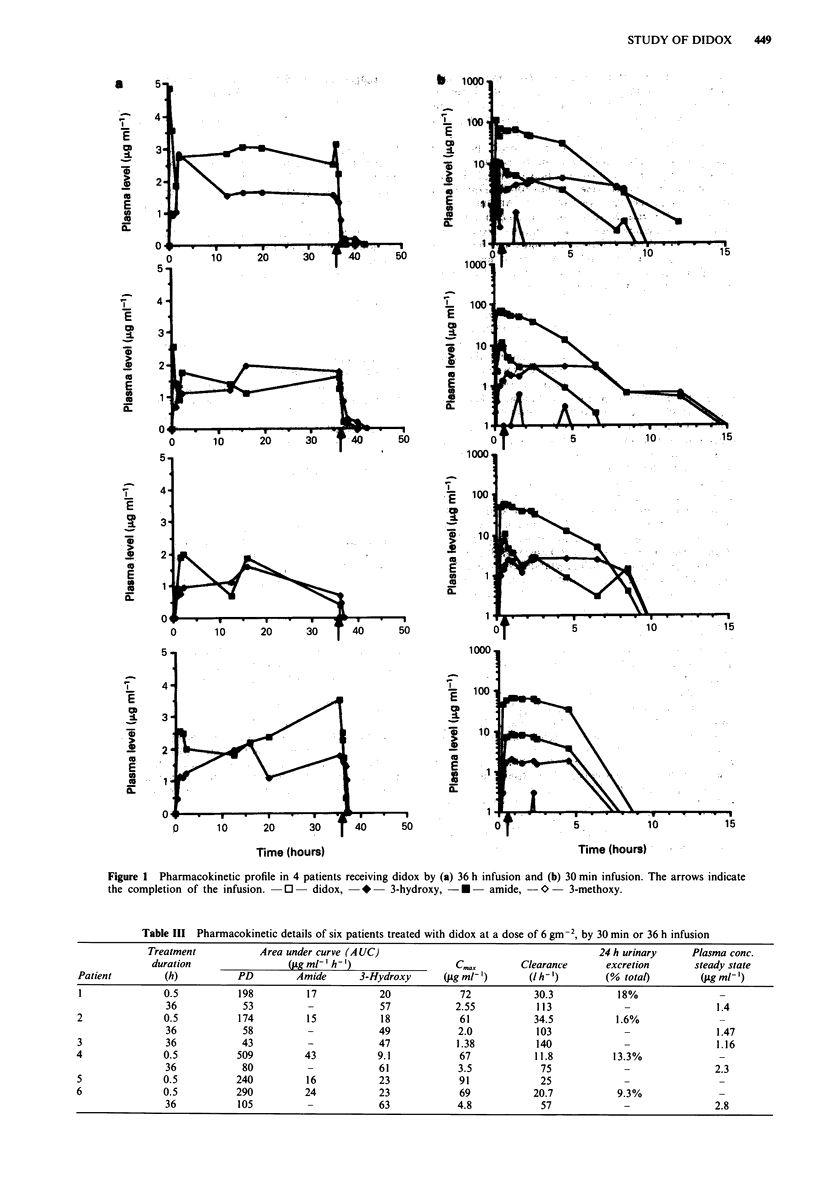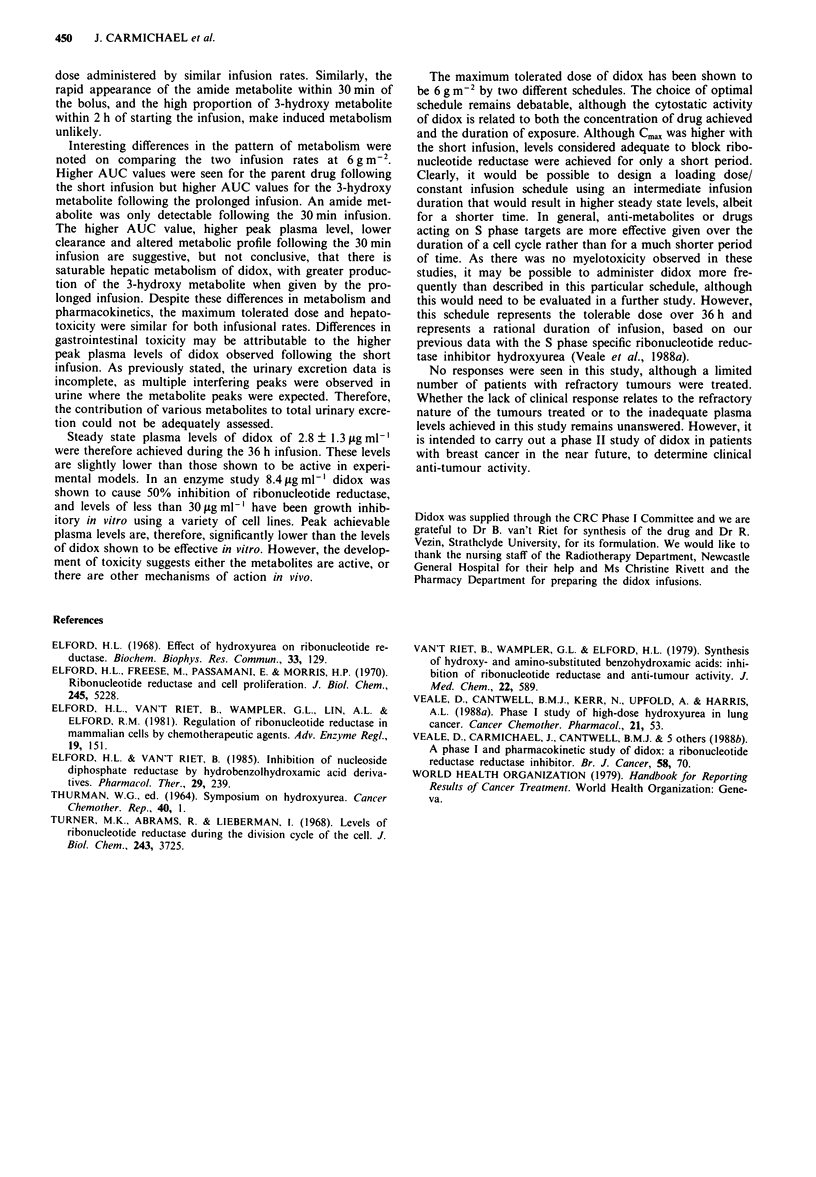# A phase I and pharmacokinetic study of didox administered by 36 hour infusion. The Cancer Research Campaign Phase I/II Clinical Trials Committee.

**DOI:** 10.1038/bjc.1990.98

**Published:** 1990-03

**Authors:** J. Carmichael, B. M. Cantwell, K. A. Mannix, D. Veale, H. L. Elford, R. Blackie, D. J. Kerr, S. B. Kaye, A. L. Harris

**Affiliations:** ICRF Department of Clinical Oncology, Churchill Hospital, Headington, Oxford, UK.

## Abstract

Twelve patients were treated with didox, a new ribonucleotide reductase inhibitor, by 36 h infusion. The maximum tolerated dose was 6 g m-2, above which dose-limiting hepatic toxicity was observed. Patient tolerance was significantly better using the 36 h infusion compared to patients receiving the drug by a 30 min infusion; in particular, there were no reports of nausea or vomiting. No responses were seen in these patients. Detailed pharmacokinetics were performed at 6 g m-2 comparing the 36 h and 30 min infusions in four patients. Parent drug AUC values were lower for the 36 h infusion, 67.8 micrograms ml-1 h-1 compared to 232 micrograms ml-1 h-1 for the 30 min infusion. AUC values for the 3-hydroxy metabolite were much higher following the 36 h infusion: 55.4 compared to 18.6 micrograms ml-1 h-1. In contrast, the amide metabolite was not detected following the 36 h infusion, but AUC values of 23 micrograms ml-1 h-1 were seen after the 30 min infusion. The mean peak plasma level was 72 micrograms ml-1 following 6 g m-2 given by a 30 min infusion compared to 2.8 micrograms ml-1 following the prolonged infusion. Clearance was higher following the 36 h infusion: 97.6 versus 24.4 l h-1.


Br. J. Cancer (1990), 61, 447–450 © Macmillan Press Ltd., 1990

### A phase I and pharmacokinetic study of didox administered by 36 hour infusion

J. Carmichael[1], B.M.J. Cantwell[2], K.A. Mannix[2], D. Veale[3], H.L. Elford[4], R. Blackie[5], D.J. Kerr[5], S.B. Kaye[5] & A.L. Harris[1] on behalf of the Cancer Research Campaign Phase I/II Clinical Trials Committee

[1]*ICRF Department of Clinical Oncology, Churchill Hospital, Headington, Oxford OX3 7LJ, UK;* [2]*University Department of Clinical Oncology, Newcastle General Hospital, Westgate Road, Newcastle upon Tyne NE4 6BE, UK;* [3]*Department of Respiratory Medicine, Regional Cardiothoracic Centre, Freeman Hospital, Newcastle upon Tyne NE2 2DN, UK;* [4]*Molecules for Health Inc., 3313 Gloucester Road, Richmond, Virginia 23227, USA; and* [5]*CRC Department of Medical Oncology, Western Infirmary, Glasgow G11 6NT, UK.*

**Summary** Twelve patients were treated with didox, a new ribonucleotide reductase inhibitor, by 36 h infusion. The maximum tolerated dose was 6 g m$^{-2}$, above which dose-limiting hepatic toxicity was observed. Patient tolerance was significantly better using the 36 h infusion compared to patients receiving the drug by a 30 min infusion; in particular, there were no reports of nausea or vomiting. No responses were seen in these patients. Detailed pharmacokinetics were performed at 6 g m$^{-2}$ comparing the 36 h and 30 min infusions in four patients. Parent drug AUC values were lower for the 36 h infusion, 67.8 μg ml$^{-1}$ h$^{-1}$ compared to 232 μg ml$^{-1}$ h$^{-1}$ for the 30 min infusion. AUC values for the 3-hydroxy metabolite were much higher following the 36 h infusion: 55.4 compared to 18.6 μg ml$^{-1}$ h$^{-1}$. In contrast, the amide metabolite was not detected following the 36 h infusion, but AUC values of 23 μg ml$^{-1}$ h$^{-1}$ were seen after the 30 min infusion. The mean peak plasma level was 72 μg ml$^{-1}$ following 6 g m$^{-2}$ given by a 30 min infusion compared to 2.8 μg ml$^{-1}$ following the prolonged infusion. Clearance was higher following the 36 h infusion: 97.6 versus 24.4 l h$^{-1}$.

The enzyme ribonucleotide reductase provides an excellent target for anti-cancer drugs in view of the importance of the production of deoxyribonucleotides for DNA synthesis (Elford *et al.*, 1981). The enzyme is known to have low activity in resting cells, and increasing with proliferation (Elford *et al.*, 1970), with the level of the enzyme correlated with the rate of replication (Turner *et al.*, 1968).

Hydroxyurea is a specific inhibitor of ribonucleotide reductase (Thurman, 1964) and is the only inhibitor available clinically at present, although it is a relatively weak inhibitor of the enzyme *in vitro* (Elford, 1968). Recently, many hydroxyurea analogues have been tested *in vivo* and *in vitro* with didox (*N*, 3-4 trihydroxybenzamide) exhibiting activity in L1210 leukaemia bearing mice (van't Riet *et al.*, 1979) and in the NCI tumour panel (Elford & van't Riet, 1985).

Didox causes greater inhibition of the target enzyme (Elford *et al.*, 1979) and in view of its *in vivo* activity was entered in phase I evaluation, as part of a co-ordinated programme under the aegis of the Cancer Research Campaign Phase I/II Clinical Trials Committee. It was initially administered by intravenous infusion over 30 min (Veale *et al.*, 1988*b*). Dose limiting toxicity was predominantly hepatic in these patients and was seen at doses of 7 g m$^{-2}$ and above. However, at doses greater than 2.3 g m$^{-2}$ significant gastrointestinal toxicity was observed, with severe nausea, vomiting and diarrhoea seen in some patients. The recommended maximum tolerated dose was therefore 6 g m$^{-2}$. Hydroxyurea has been given safely by infusion over 24–36 h infusion (Veale *et al.*, 1988*a*), and didox was therefore administered by 36 h infusion in a phase I study in an attempt to cause more prolonged inhibition of ribonucleotide reductase. Detailed pharmacokinetics were performed at 6 g m$^{-2}$ comparing both 36 h and 30 min infusions.

### Patients and methods

Patients with histologically proven metastatic malignant disease who had either failed first line chemotherapy or for whom no conventional treatment existed, were entered into this study. All patients had normal renal and liver function and were of good performance status (ECOG 0–2; WHO, 1979). Patients had received no other cytotoxic chemotherapy for 1 month before the study.

As the maximum tolerated dose for the slow i.v. injection was 6 g m$^{-2}$ (Veale *et al.*, 1988*b*), infusional didox treatment commenced at a dose of 2.5 g m$^{-2}$. The dose was then increased to 5 g m$^{-2}$ and subsequently by 1 g m$^{-2}$ increments. Didox was dissolved in 3 litres of 0.5 N dextrose saline and given as a continuous infusion over 36 h. For the comparative pharmacokinetic study, slow intravenous injections were given at a dose of 6 g m$^{-2}$ in 500 ml 0.5 N dextrose saline over 30 min, with patients randomly receiving either the slow i.v. injection or 36 h infusion on alternate courses. Patients were treated every 3 weeks, with a minimum of three patients treated at each dose increment.

### *Pharmacokinetics*

Blood samples were taken from an indwelling catheter at the following times: (a) 30 min infusion: pre-treatment, 15 and 30 min, then 5, 15, 30, 60 min, 2, 4, 6, 8 and 24 h post-infusion; (b) 36 h infusion: pre-treatment, 0.5, 1, 1.5, 2, 6, 12, 24, 36 h, then 5, 15, 30, 60 min, 2, 4, 6 and 8 h post-infusion.

Urine was collected and aliquoted pre-treatment and subsequently every 6 h during and post-treatment up to 12 h following completion of treatment.

### *Analytical methods*

Didox levels were measured in plasma and urine by HPLC using a Beckman/Altex 100A pump and stainless steel column (15 cm × 0.46 cm) packed with a μ-Bondapack C18, 10 μm particle size, as previously described (Veale *et al.*, 1988). Metabolites were identified using standards supplied by Elford. In order to resolve didox and its metabolites from interfering substances in plasma the following step-gradient system was employed: (1) 0.1 M sodium phosphate pH 6.0 for 2 min; (2) as above + 2% acetonitrile pH 6.0 for 2 min; (3) as above + 5% acetonitrile pH 6.0 for 4 min; (4) as above + 10% acetonitrile pH 6.0 for 0.5 min; (5) as above + 20% acetonitrile pH 6.0 for 0.5 min; (6) as above + 30% acetonitrile pH 6.0 for 10 min. The column was equilibrated with

Correspondence: A.L. Harris.
Received 28 November 1988; and in revised form 18 September 1989.

buffer 1 for 7 min before loading the next sample.

Retention times of 4 min, 7.78 min, 9.5 min, 10.45 min and 12.86 min were observed for didox, amide, 3-OH, 3-MeO and I.S. respectively. Extraction efficiency was 80%, 82%, 95%, 94% for didox, amide, 3-hydroxy and 3-methoxy metabolites, with a detection limit of 100 ng for each compound. Urine samples were injected directly onto the column and eluted isocratically with 0.1 M sodium phosphate pH 6.0. The resolution of metabolites in urine was not possible due to the large number of interfering substances.

The area under the plasma concentration–time curve (AUC) was calculated by the log trapezoidal rule with extrapolation to infinity. As the drug was proven subsequently to have non-linear pharmacokinetics, it was not deemed logical to fit the data to linear compartmental models. Drug clearance was calculated using the expression:

$$\text{Clearance} = \frac{\text{dose}}{\text{AUC}}$$

### Results

Patient details are shown in Table I. In the 36 h infusion dose escalation study, three patients received 2.5 g $m^{-2}$, three 5 g $m^{-2}$, ten 6 g $m^{-2}$, and two 7 g $m^{-2}$ of didox. In addition, five patients were treated at 6 g $m^{-2}$ as a 30 min infusion as part of the comparative pharmacokinetic study. No responses were seen at any dose level.

Details of toxicity are shown in Table II. No toxicity was seen with the infusion up to 6 g $m^{-2}$. At 6 g $m^{-2}$ grade 1 hepatotoxicity was seen in two of 10 patients and grade 2 toxicity in one patient, but these abnormalities were rapidly reversible. At 7 g $m^{-2}$ grade 1 hepatotoxicity was seen in one patient and grade 3 hepatotoxicity in the other patient. This toxicity was considered dose-limiting, with the maximum tolerated dose therefore 6 g $m^{-2}$. Gastrointestinal toxicity was absent up to a dose of 7 g $m^{-2}$. Side-effects following the 30 min infusion are shown in Table II, relating to the 6 g $m^{-2}$ dose. Gastrointestinal toxicity was severe, with grade 3 nausea and vomiting in three of five patients, and minor hepatotoxicity observed in three patients.

Four patients received two courses at 6 g $m^{-2}$, one by 30 min and the other as a 36 h infusion. Pharmacokinetic profiles for these patients illustrated in Figure 1, with the various parameters summarised in Table III. Two other patients received only one course of didox (6 g $m^{-2}$), one as a 30 min and the other as a 36 h infusion. The mean AUC value (± s.e.m.) for the parent drug following the 30 min infusion was significantly higher (282 ± 60 versus 68 ± 11 μg $ml^{-1}$ $h^{-1}$). In contrast, AUC levels for the 3-hydroxy metabolite of didox were significantly higher following the prolonged infusion (55.4 ± 3.2 versus 18.6 ± 2.6 μg $ml^{-1}$ $h^{-1}$). Significant levels of the amide metabolite of didox were observed following the 30 min infusion, but this metabolite was not detected following the prolonged infusion. End of infusion peak plasma didox levels of 72 ± 5 and 2.8 ± 0.6 μg $ml^{-1}$ were seen following the short and prolonged infusions respectively, with steady state didox levels of 1.8 μg $ml^{-1}$ achieved during the latter. Clearance values were much higher following the prolonged infusion (98 ± 14 versus 24 ± 4 l $h^{-1}$). Following the 30 min infusion 12.4% of the didox was excreted unchanged in the urine within 24 h, with only 5% of unchanged drug recovered following the prolonged infusion. Interfering peaks were observed in the urine where the didox metabolites were expected, therefore the level of urinary excretion of didox metabolites could not be determined.

**Table I** Patient characteristics

| | |
|---|---|
| Number of patients | 12 |
| Male : female | 5:7 |
| Age (range) | 49.8 (39–72) |
| Previous chemotherapy | 11 |
| Histology | |
| Sarcoma | 3 |
| Melanoma | 3 |
| Colon | 2 |
| Mesothelioma | 1 |
| Breast | 1 |
| Small cell lung cancer | 1 |
| Ovary | 1 |

**Table II** Toxicity of didox administered either by a 30 min or 36 h infusion

| Symptom | Drug dose (mg $m^{-2}$) | No. pts | Toxicity (WHO grade) 1 | 2 | 3 | 4 |
|---|---|---|---|---|---|---|
| *36 h infusion* | | | | | | |
| Nausea and vomiting | 2500 | 3 | – | – | – | – |
| | 5000 | 3 | – | – | – | – |
| | 6000 | 10 | – | – | – | – |
| | 7000 | 2 | – | – | 1 | – |
| LFTs | | | | | | |
| Bilirubin | < 5000 | 6 | – | – | – | – |
| | 6000 | 10 | – | 1 | – | – |
| | 7000 | 2 | – | – | 1 | – |
| AST | < 5000 | 6 | – | – | – | – |
| | 6000 | 10 | 3 | – | – | – |
| | 7000 | 2 | 1 | – | – | 1 |
| Alk. phos. | < 5000 | 6 | – | – | – | – |
| | 6000 | 10 | 2 | 1 | – | – |
| | 7000 | 2 | – | 1 | 1 | – |
| Pain | 7000 | 2 | – | – | 1 | – |
| *30 min infusion* | | | | | | |
| Nausea and vomiting | 6000 | 5 | 1 | – | 3 | – |
| LFTs | | | | | | |
| Bilirubin | 6000 | 5 | 1 | – | – | – |
| AST | 6000 | 5 | 2 | 1 | – | – |
| Alk. phos. | 6000 | 5 | 2 | 1 | – | – |

### Discussion

A phase 1 study of didox, administered as a 36 h infusion, was performed in 12 patients. Toxicity was minimal up to 6 g $m^{-2}$ when minor hepatotoxicity was observed, with severe hepatotoxicity noted in one of the two patients treated at 7 g $m^{-2}$. No myelosuppression was seen at any dose level. Gastrointestinal toxicity was severe with the short injection at 6 g $m^{-2}$, although minor hepatotoxicity was also noted. The recommended maximum tolerated dose for both routes of administration was 6 g $m^{-2}$, although the toxicity profile was different for the two modes of administration. Despite marked differences in pharmacokinetics and drug metabolism between the injection and infusion, the maximum tolerated dose of the drug was the same, with hepatotoxicity dose-limiting in both. However, the prolonged infusion was significantly better tolerated and in particular gastrointestinal toxicity was rare.

In a previous study (Veale et al., 1988b) didox was given as a short injection in doses up to 10 g $m^{-2}$. Pharmacokinetics were performed at 1,728 mg $m^{-2}$ in that study, showing α-half life of 5.2 min, β-half life of 41.3 min and clearance of 42.6 ± 11.4 l $h^{-1}$. Although the infusion rates were different in both studies (short infusion versus 30 min) this is unlikely to produce a difference in pharmacokinetics and was accounted for in calculations. Thus clearance showed a marked difference between the two different doses. It is possible that the pharmacokinetics of didox are non-linear as drug clearance is dose-dependent. The ideal way to test this hypothesis is by performing pharmacokinetic studies at each dose level for a schedule, but this was not performed in the present study.

Clearance was also schedule-dependent. It is possible that the increase in clearance of the parent drug seen on increasing the duration of infusion from 0.5 to 36 h could be related to induction of its own metabolism, but this would not explain the reduction in clearance caused by increasing drug

**Figure 1** Pharmacokinetic profile in 4 patients receiving didox by (**a**) 36 h infusion and (**b**) 30 min infusion. The arrows indicate the completion of the infusion. —□— didox, —◆— 3-hydroxy, —■— amide, —◇— 3-methoxy.

**Table III** Pharmacokinetic details of six patients treated with didox at a dose of 6 gm$^{-2}$, by 30 min or 36 h infusion

| *Patient* | *Treatment duration (h)* | *Area under curve (AUC) (μg ml⁻¹ h⁻¹)* PD | *Amide* | *3-Hydroxy* | $C_{max}$ *(μg ml⁻¹)* | *Clearance (l h⁻¹)* | *24 h urinary excretion (% total)* | *Plasma conc. steady state (μg ml⁻¹)* |
|---|---|---|---|---|---|---|---|---|
| 1 | 0.5 | 198 | 17 | 20 | 72 | 30.3 | 18% | – |
| | 36 | 53 | – | 57 | 2.55 | 113 | – | 1.4 |
| 2 | 0.5 | 174 | 15 | 18 | 61 | 34.5 | 1.6% | – |
| | 36 | 58 | – | 49 | 2.0 | 103 | – | 1.47 |
| 3 | 36 | 43 | – | 47 | 1.38 | 140 | – | 1.16 |
| 4 | 0.5 | 509 | 43 | 9.1 | 67 | 11.8 | 13.3% | – |
| | 36 | 80 | – | 61 | 3.5 | 75 | – | 2.3 |
| 5 | 0.5 | 240 | 16 | 23 | 91 | 25 | – | – |
| 6 | 0.5 | 290 | 24 | 23 | 69 | 20.7 | 9.3% | – |
| | 36 | 105 | – | 63 | 4.8 | 57 | – | 2.8 |

dose administered by similar infusion rates. Similarly, the rapid appearance of the amide metabolite within 30 min of the bolus, and the high proportion of 3-hydroxy metabolite within 2 h of starting the infusion, make induced metabolism unlikely.

Interesting differences in the pattern of metabolism were noted on comparing the two infusion rates at $6\,g\,m^{-2}$. Higher AUC values were seen for the parent drug following the short infusion but higher AUC values for the 3-hydroxy metabolite following the prolonged infusion. An amide metabolite was only detectable following the 30 min infusion. The higher AUC value, higher peak plasma level, lower clearance and altered metabolic profile following the 30 min infusion are suggestive, but not conclusive, that there is saturable hepatic metabolism of didox, with greater production of the 3-hydroxy metabolite when given by the prolonged infusion. Despite these differences in metabolism and pharmacokinetics, the maximum tolerated dose and hepatotoxicity were similar for both infusional rates. Differences in gastrointestinal toxicity may be attributable to the higher peak plasma levels of didox observed following the short infusion. As previously stated, the urinary excretion data is incomplete, as multiple interfering peaks were observed in urine where the metabolite peaks were expected. Therefore, the contribution of various metabolites to total urinary excretion could not be adequately assessed.

Steady state plasma levels of didox of $2.8 \pm 1.3\,\mu g\,ml^{-1}$ were therefore achieved during the 36 h infusion. These levels are slightly lower than those shown to be active in experimental models. In an enzyme study $8.4\,\mu g\,ml^{-1}$ didox was shown to cause 50% inhibition of ribonucleotide reductase, and levels of less than $30\,\mu g\,ml^{-1}$ have been growth inhibitory *in vitro* using a variety of cell lines. Peak achievable plasma levels are, therefore, significantly lower than the levels of didox shown to be effective *in vitro*. However, the development of toxicity suggests either the metabolites are active, or there are other mechanisms of action *in vivo*.

The maximum tolerated dose of didox has been shown to be $6\,g\,m^{-2}$ by two different schedules. The choice of optimal schedule remains debatable, although the cytostatic activity of didox is related to both the concentration of drug achieved and the duration of exposure. Although $C_{max}$ was higher with the short infusion, levels considered adequate to block ribonucleotide reductase were achieved for only a short period. Clearly, it would be possible to design a loading dose/constant infusion schedule using an intermediate infusion duration that would result in higher steady state levels, albeit for a shorter time. In general, anti-metabolites or drugs acting on S phase targets are more effective given over the duration of a cell cycle rather than for a much shorter period of time. As there was no myelotoxicity observed in these studies, it may be possible to administer didox more frequently than described in this particular schedule, although this would need to be evaluated in a further study. However, this schedule represents the tolerable dose over 36 h and represents a rational duration of infusion, based on our previous data with the S phase specific ribonucleotide reductase inhibitor hydroxyurea (Veale *et al.*, 1988*a*).

No responses were seen in this study, although a limited number of patients with refractory tumours were treated. Whether the lack of clinical response relates to the refractory nature of the tumours treated or to the inadequate plasma levels achieved in this study remains unanswered. However, it is intended to carry out a phase II study of didox in patients with breast cancer in the near future, to determine clinical anti-tumour activity.

Didox was supplied through the CRC Phase I Committee and we are grateful to Dr B. van't Riet for synthesis of the drug and Dr R. Vezin, Strathclyde University, for its formulation. We would like to thank the nursing staff of the Radiotherapy Department, Newcastle General Hospital for their help and Ms Christine Rivett and the Pharmacy Department for preparing the didox infusions.

### References

ELFORD, H.L. (1968). Effect of hydroxyurea on ribonucleotide reductase. *Biochem. Biophys. Res. Commun.*, **33**, 129.

ELFORD, H.L., FREESE, M., PASSAMANI, E. & MORRIS, H.P. (1970). Ribonucleotide reductase and cell proliferation. *J. Biol. Chem.*, **245**, 5228.

ELFORD, H.L., VAN'T RIET, B., WAMPLER, G.L., LIN, A.L. & ELFORD, R.M. (1981). Regulation of ribonucleotide reductase in mammalian cells by chemotherapeutic agents. *Adv. Enzyme Regl.*, **19**, 151.

ELFORD, H.L. & VAN'T RIET, B. (1985). Inhibition of nucleoside diphosphate reductase by hydrobenzolhydroxamic acid derivatives. *Pharmacol. Ther.*, **29**, 239.

THURMAN, W.G., ed. (1964). Symposium on hydroxyurea. *Cancer Chemother. Rep.*, **40**, 1.

TURNER, M.K., ABRAMS, R. & LIEBERMAN, I. (1968). Levels of ribonucleotide reductase during the division cycle of the cell. *J. Biol. Chem.*, **243**, 3725.

VAN'T RIET, B., WAMPLER, G.L. & ELFORD, H.L. (1979). Synthesis of hydroxy- and amino-substituted benzohydroxamic acids: inhibition of ribonucleotide reductase and anti-tumour activity. *J. Med. Chem.*, **22**, 589.

VEALE, D., CANTWELL, B.M.J., KERR, N., UPFOLD, A. & HARRIS, A.L. (1988*a*). Phase I study of high-dose hydroxyurea in lung cancer. *Cancer Chemother. Pharmacol.*, **21**, 53.

VEALE, D., CARMICHAEL, J., CANTWELL, B.M.J. & 5 others (1988*b*). A phase I and pharmacokinetic study of didox: a ribonucleotide reductase reductase inhibitor. *Br. J. Cancer*, **58**, 70.

WORLD HEALTH ORGANIZATION (1979). *Handbook for Reporting Results of Cancer Treatment*. World Health Organization: Geneva.